# Implementation Strategies to Enhance the Implementation of eHealth Programs for Patients With Chronic Illnesses: Realist Systematic Review

**DOI:** 10.2196/14255

**Published:** 2019-09-27

**Authors:** Cecilie Varsi, Lise Solberg Nes, Olöf Birna Kristjansdottir, Saskia M Kelders, Una Stenberg, Heidi Andersen Zangi, Elin Børøsund, Karen Elizabeth Weiss, Audun Stubhaug, Rikke Aune Asbjørnsen, Marianne Westeng, Marte Ødegaard, Hilde Eide

**Affiliations:** 1 Center for Shared Decision Making and Collaborative Care Research Division of Medicine Oslo University Hospital Oslo Norway; 2 Institute of Clinical Medicine Faculty of Medicine University of Oslo Oslo Norway; 3 Department of Psychiatry and Psychology Mayo Clinic Rochester, MN United States; 4 Norwegian National Advisory Unit on Learning and Mastery in Health Oslo University Hospital Oslo Norway; 5 Center for eHealth and Wellbeing Research Department of Psychology, Health and Technology University of Twente Enschede Netherlands; 6 Optentia Research Focus Area North-West University Vanderbijlpark South Africa; 7 National Advisory Unit on Rehabilitation in Rheumatology Department of Rheumatology Diakonhjemmet Hospital Oslo Norway; 8 Faculty of Health VID Specialized University Oslo Norway; 9 Department of Anesthesiology and Pain Medicine School of Medicine University of Washington Seattle, WA United States; 10 Regional Advisory Unit on Pain Oslo University Hospital Oslo Norway; 11 Department of Pain Management and Research Division of Emergencies and Critical Care Oslo University Hospital Oslo Norway; 12 Department of Research and Innovation Vestfold Hospital Trust Tønsberg Norway; 13 University of Oslo Library University of Oslo Oslo Norway; 14 Science Centre Health and Technology University of South-Eastern Norway Drammen Norway

**Keywords:** chronic illness, eHealth, implementation, implementation strategies, implementation outcomes, realist review

## Abstract

**Background:**

There is growing evidence of the positive effects of electronic health (eHealth) interventions for patients with chronic illness, but implementation of such interventions into practice is challenging. Implementation strategies that potentially impact implementation outcomes and implementation success have been identified. Which strategies are actually used in the implementation of eHealth interventions for patients with chronic illness and which ones are the most effective is unclear.

**Objective:**

This systematic realist review aimed to summarize evidence from empirical studies regarding (1) which implementation strategies are used when implementing eHealth interventions for patients with chronic illnesses living at home, (2) implementation outcomes, and (3) the relationship between implementation strategies, implementation outcomes, and degree of implementation success.

**Methods:**

A systematic literature search was performed in the electronic databases MEDLINE, Embase, PsycINFO, Scopus, Allied and Complementary Medicine Database, Cumulative Index to Nursing and Allied Health Literature, and Cochrane Library. Studies were included if they described implementation strategies used to support the integration of eHealth interventions into practice. Implementation strategies were categorized according to 9 categories defined by the Expert Recommendations for Implementing Change project: (1) engage consumers, (2) use evaluative and iterative strategies, (3) change infrastructure, (4) adapt and tailor to the context, (5) develop stakeholder interrelationships, (6) use financial strategies, (7) support clinicians, (8) provide interactive assistance, and (9) train and educate stakeholders. Implementation outcomes were extracted according to the implementation outcome framework by Proctor and colleagues: (1) acceptability, (2) adoption, (3) appropriateness, (4) cost, (5) feasibility, (6) fidelity, (7) penetration, and (8) sustainability. Implementation success was extracted according to the study authors’ own evaluation of implementation success in relation to the used implementation strategies.

**Results:**

The implementation strategies management support and engagement, internal and external facilitation, training, and audit and feedback were directly related to implementation success in several studies. No clear relationship was found between the number of implementation strategies used and implementation success.

**Conclusions:**

This is the first review examining implementation strategies, implementation outcomes, and implementation success of studies reporting the implementation of eHealth programs for patients with chronic illnesses living at home. The review indicates that internal and external facilitation, audit and feedback, management support, and training of clinicians are of importance for eHealth implementation. The review also points to the lack of eHealth studies that report implementation strategies in a comprehensive way and highlights the need to design robust studies focusing on implementation strategies in the future.

**Trial Registration:**

PROSPERO CRD42018085539; https://www.crd.york.ac.uk/prospero/display_record.php?RecordID=85539

## Introduction

Electronic health (eHealth), defined as “health services and information delivered or enhanced through the Internet and related technologies” [1], has great potential for persons with chronic or long-term illnesses. For example, eHealth provides options for self-management, patient-provider communication, monitoring, and shared decision making [2-5]. A growing body of evidence indicates positive effects of eHealth services on patient health outcomes [6-9]. For example, telehealth is regarded as a safe option for delivery of self-management support [10], and internet-delivered cognitive behavioral therapy (ICBT) has shown promising results as an alternative to traditional face-to-face interventions among persons with chronic health illnesses [3]. Similarly, studies indicate that eHealth services can be effective in reducing hospital admissions for patients with chronic illnesses such as chronic obstructive pulmonary disease [11] and reducing symptoms of anxiety and depression [12] and may contribute to successful self-management of chronic pain [13]. Moreover, patients using eHealth services have reported high levels of acceptability and satisfaction [11,14], and health care providers have described clinical benefits from eHealth services [2]. Understanding more about the implementation of eHealth services for patients with chronic conditions, a large patient group with unpredictable disease trajectories and the need for coordinated long-term multidisciplinary follow-up, would be beneficial and could lead to successful implementation in other areas.

Even with a growing number of eHealth programs, many of which have shown promising results [15], the actual implementation of such programs into everyday use in clinical practice has proven to be challenging [16,17]. The implementation process can be demanding and requires significant effort to succeed [18]. The phase between the organizational decision to adopt an eHealth program and the health care providers’ routine use of that program is multifaceted and complex [4,15,18]. Implementation strategies, defined as “a systematic intervention process to adopt and integrate evidence-based health innovations into usual care” [19], can aid the implementation of eHealth programs into practice [18-20]. Implementation strategies constitute the how-to component of changing health care practice [20], and a number of known implementation strategies can possibly impact implementation success [19,21,22]. When implementation is initiated in a clinical health care setting, the use of implementation strategies refers to the concrete activities taken to make patients and health care providers start and maintain use of new evidence within the clinical setting. Implementation strategies are often part of an implementation plan, which describes what will be implemented, to whom, how and when, with the implementation strategies constituting the how-to in the plan. The implementation strategies can include a wide range of activities directed toward different stakeholders (eg, involvement of health care providers and patients, training and follow-up in the delivery of the clinical intervention, leadership engagement and internal and external support) [22]. The implementation strategies can be used as standalone (discrete) strategies or as a combination of strategies (multifaceted) [23]. Even though the research on implementation strategies is still in its infancy, there is a growing recognition that implementation will not happen automatically and that use of implementation strategies can be effective, particularly as they target those intending to use the new evidence directly [23,24].

Despite existing implementation strategy taxonomies and implementation process models (ie, practical guidance in the use of implementation strategies to facilitate implementation) [25], and the fact that some organizations have developed a set of implementation strategies for use in their own implementation processes [26], there is still limited understanding regarding which strategies to use and the relative importance of these strategies when promoting use of evidence-based interventions in clinical practice [22,27]. Notably, Greenhalgh and colleagues [28,29], who recently developed and tested a framework for nonadoption, abandonment, scale-up, spread, and sustainability (NASSS) of health and care technologies, argue that technology implementation will not succeed until the complexities of multiple, interacting domains (eg, the illness, the technology, the organization, and the implementation process) are taken into account and addressed. With the exception of a few initiatives such as the one taken by the Greenhalgh group, little emphasis has been placed on the planning of and reporting on implementation strategies related to the implementation of eHealth interventions into practice [30,31]. Research literature has summarized different aspects of eHealth implementation [18], including barriers and facilitators [32-34], frontline staff acceptance of eHealth technologies [35], patient recruitment strategies [36], and eHealth implementation in rural areas [31]. However, the empirical research literature on strategies for eHealth implementation has not yet been reviewed or summarized. Also, the relationship between implementation strategies, implementation outcomes, and implementation success is rarely adequately described.

Implementation outcomes can be measured by means of various methods (eg, qualitative, quantitative, mixed), and the success of the implementation effort can be evaluated on the basis of implementation outcomes [37]. When seeking to understand implementation outcomes, researchers have stated that the relative importance of each single outcome measurement may vary in importance depending on stakeholders and may have different consequences depending on setting [37]. This indicates that implementation success is not necessarily derived directly from the implementation outcome measurements. Therefore, assessment of implementation success in addition to implementation outcomes can, as pointed out by Proctor and colleagues [37], play an important role in understanding and assessing the success of the implementation effort.

This project sought to further research and gain knowledge in this area through a systematic realist review. The realist review approach involves identifying how and why interventions work (or fail to work) in different contexts and examines the links between context, mechanisms, and outcomes [38]. Unlike classical systematic reviews, realist reviews focus not only on if the program works but also on how, why, and for whom [38]. The approach is often described as “what works for whom under what circumstances and why.” As noted by Rycroft-Malone and colleagues [39], the realist review method is especially suited when conducting reviews on implementation, due to implementation processes’ complex, multifaceted nature and the limited understanding of their mechanisms of action [39]. This systematic realist review aimed to summarize evidence from empirical studies regarding (1) which implementation strategies were used when implementing eHealth interventions for patients with chronic illnesses living at home; (2) which implementation outcomes were achieved; and (3) the relationship between implementation strategies, implementation outcomes, and degree of implementation success.

## Methods

### Overview

A systematic realist review, by means of an aggregative approach using predefined concepts (ie, implementation strategies and implementation outcomes) [40] was considered suitable to provide an explanatory analysis focusing on which implementation strategies were used, in what circumstances, how, and leading to which implementation outcomes. In addition, as an evaluation of the reported implementation outcomes, the degree of implementation success was summarized qualitatively based on the study authors’ own definition. This review focused on the implementation of eHealth programs used by patients with chronic illness in their own homes. See Table 1 for details, key terms and definitions. The protocol for this realist systematic review has been registered and published in the Prospective Register of Systematic Reviews (PROSPERO; CRD42018085539).

**Table 1:** Key terms and their definitions.

Term	Definition
eHealth	Health services and information delivered or enhanced through the internet and related technologies [1]. Including but not limited to: mHealth (mobile health): health practice supported by mobile devices [41] Telehealth: using telecommunications and virtual technology to deliver health care outside of traditional health care facilities [42] Patient portals (secure online websites that give patients access to personal health information) [43] For inclusion in this review, the eHealth program had to have patients/clients in their own homes as the primary users, optionally with support or involvement from health care providers. In this publication, the collective term eHealth is used unless a more specific definition is considered of essence.
Implementation	Process of putting to use or integrating evidence-based interventions within a setting [44].
Implementation strategy	Systematic intervention process to adopt and integrate evidence-based health innovations into usual care [19]. The Expert Recommendations for Implementing Change project has defined and sorted implementation strategies into a taxonomy consisting of the following categories: (1) engage consumers, (2) use evaluative and iterative strategies, (3) change infrastructure, (4) adapt and tailor to the context, (5) develop stakeholder interrelationships, (6) use financial strategies, (7) support clinicians, (8) provide interactive assistance, and (9) train and educate stakeholders [22].
Implementation outcome	Effects of deliberate and purposive actions to implement new treatments, practices, and services [37]. Proctor and colleagues [37] have defined and sorted implementation outcomes into the implementation outcome framework consisting of the following terms: (1) acceptability, (2) adoption, (3) appropriateness, (4) costs, (5) feasibility, (6) fidelity, (7) penetration, and (8) sustainability.

### Literature Search

A systematic literature search was performed by the librarian (MØ) in the electronic databases MEDLINE, Embase, PsycInfo and Allied and Complementary Medicine Database (Ovid), Cumulative Index to Nursing and Allied Health Literature (EBSCOhost), Scopus, and Cochrane Library. The search terms were developed by the first author (CV) and the librarian (MØ) using a combination of keywords and database-specific headings and covered the period from January 1, 2006, to October 4, 2018. The starting point for the review period was set to the year of the first issue of the journal Implementation Science (2006), since there was a pronounced focus on implementation from that point, although some researchers had been working within this field earlier. The basic search strategy (Multimedia Appendix 1) was modified for use in each database. Additional studies were detected based on references and citations in the included studies.

### Criteria for Considering Studies for the Review

Inclusion criteria for studies in the review were the reporting of implementation strategies used in the implementation of eHealth programs seeking to support adults with chronic illness in their own homes. Studies were included only if they provided a description of the implementation strategies they had used. Studies were, for example, excluded if they only mentioned training had been conducted or management had been involved without any further description of the content of the training or management engagement.

The following illnesses were included: chronic disease, arthritis, chronic pain, chronic obstructive pulmonary disease, obesity, diabetes mellitus, and mental disorder. Empirical studies in English, Dutch, and Scandinavian languages published in peer-reviewed journals were included. All study designs were included. Literature reviews, meta-analyses, theoretical articles, book chapters, editorials, study protocols, dissertations, studies published in abstract form only, and duplicates were excluded. eHealth programs involving primarily children, adolescents, and family care givers or solely for health care providers were excluded.

### Study Selection Process

All titles and abstracts were reviewed by the first author (CV). Irrelevant publications (eg, studies focusing on non-eHealth programs) were excluded. Next, two of the authors (CV and one of the coauthors) independently reviewed titles and abstracts using the systematic review software Covidence (Veritas Health Innovation). When the authors were in agreement, the studies were included for full-text review. When the authors were not in agreement, the first author (CV) conducted a second review and subsequently made a decision. If there was doubt, the study was selected for full-text review. Next, CV and one of the coauthors independently reviewed full-text studies separately. When the authors agreed, the studies were included. If the authors disagreed, the first author conducted a second review and subsequently made a final decision. The authors met several times during this process in order to discuss and reach agreement on the understanding of the inclusion and exclusion criteria.

### Data Extraction and Evidence Appraisal

Data were extracted using a data extraction form developed by the authors for the purpose of this review relating to the study details, country of origin, design, setting, population, demographics, intervention, implementation framework, implementation strategies, implementation outcomes, and implementation success. NVivo software version 11 (QSR International) was used to organize and facilitate the extraction. The data extraction was guided by the aims of the review, focusing on (1) implementation strategies used, (2) implementation outcomes achieved, and (3) the relationship between implementation strategies, implementation outcomes, and degree of implementation success. The identified implementation strategies were sorted according to the 9 categories defined by the Expert Recommendations for Implementing Change (ERIC) project [22]. See Table 2 for specific description of implementation strategies. The identified implementation outcomes were sorted by the 8 categories in the implementation outcome framework defined by Proctor and colleagues [37]. See Table 3 for specific description of implementation outcomes. The taxonomies of ERIC and Proctor have been successfully used by other researchers [45-47] and were used in this review. Implementation success was extracted according to the study authors’ own evaluation of implementation success in relation to the implementation strategies used, not based on a specific framework. The data extraction was conducted in two steps. First, implementation strategies, implementation outcomes, and implementation success were extracted separately. Next, these 3 sets of data were put together in a table to evaluate their interrelationships (eg, qualitatively assessing whether certain combinations were more common than others). The first author (CV) extracted data from all included studies. A second author (SMK) validated the data extraction of 25% (3/12) of the included studies.

Traditional quality assessment of the included studies in this review was not undertaken. The realist review methodology does not lean on the traditional study hierarchy assessment with the randomized controlled trials at the top, as it is acknowledged that multiple methods are needed to cover the entire picture of what works for whom and under which circumstances [38]. The relevance of the included studies was considered based on each study’s ability to answer the research questions of the review, including that the studies had provided at least a minimum description of the content of the implementation strategies used to be incorporated. Rigor was considered related to the study authors’ credibility based on the conclusions made in the included studies.

**Table 2:** Implementation strategies (adapted from Waltz and colleagues [22]).

Implementation strategies	Description
Engage consumers	Involving, preparing, and intervening with patients and the market to involve them and increase demand for the clinical innovation
Use evaluative and iterative strategies	Planning and conducting the implementation process, including activities such as make a plan, assess for readiness, identify barriers and facilitators, evaluate performance and progress, and provide audit and feedback
Change infrastructure	Changing external structures such as legislation models, as well as internal conditions such as facilities and equipment
Adapt and tailor to the context	Tailoring the innovation to meet local needs and tailoring the implementation strategies toward the identified barriers and facilitators
Develop stakeholder interrelationships	Involving relevant internal and external stakeholders to support and move the implementation process forward
Use financial strategies	Changing the patient billing systems, fee structures, reimbursement policies, research funding, and clinician incentives
Support clinicians	Supporting clinical staff performance
Provide interactive assistance	Supporting implementation issues
Train and educate stakeholders	Providing written and oral training

**Table 3:** Implementation outcomes (adapted from Proctor and colleagues [37]).

Implementation outcomes	Description
Acceptability	Perception that a given treatment, service, practice, or innovation is agreeable, palatable, or satisfactory
Adoption	Intention, initial decision, or action to try or employ an innovation or evidence-based practice
Appropriateness	Perceived fit, relevance, or compatibility of the innovation or evidence-based practice for a given practice setting, provider, or consumer and/or perceived fit of the innovation to address a particular issue or problem
Cost	Cost impact of an implementation effort (incremental or implementation cost)
Feasibility	Extent to which a new treatment or innovation can be successfully used or carried out within a given agency or setting
Fidelity	Degree to which an intervention was implemented as it was prescribed in the original protocol or intended by the program developers
Penetration	Integration of a practice within a service setting and its subsystems
Sustainability	Extent to which a newly implemented treatment is maintained or institutionalized within a service setting’s ongoing, stable operations

## Results

### Overview of Included Studies

**Figure 1:**
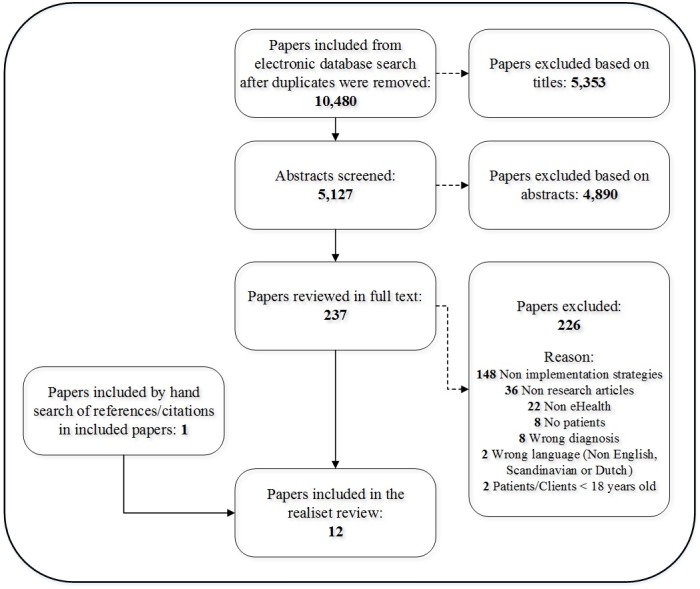
Flow diagram of the study selection process.

The search generated 10,480 unique references. From these references, 5353 were excluded based on the title alone and an additional 4890 were excluded based on the abstract. The inconsistency in terms used in the research literature on eHealth and implementation strategies led to a large number of hits on irrelevant studies. Most of these studies were therefore excluded, and 237 studies were selected for full text evaluation. Following evaluation by two independent authors (ie, the first author and one coauthor), 11 studies met all inclusion criteria and were included [48-58]. In addition, one study was included based on a manual search of references and citations in the first 11 included studies [59]. See Figure 1 for details on the study selection process.

Seven of the 12 included studies used qualitative research design [48,50,52,53,55,58,59], 2 used quantitative design in terms of surveys [51,56], and 3 used mixed-methods design [49,54,57]. Of the final 12 included studies, 2 studies were conducted in the United States [50,54], one in Canada [51], 5 in the United Kingdom [48,52,53,55,59], 2 in the Netherlands [49,56], one in Norway [58], and one in New Zealand [57]. All 12 were published in English.

Two of the 12 included studies were conducted in early phases of the implementation (ie, up to 3 months after implementation startup) [50,56]. Four studies were conducted 4 to 12 months after implementation startup, defined as middle phase [48,49,53,55]. The remaining 6 studies were conducted more than 1 year after implementation startup, defined as late phase [51,52,54,57-59], and 4 of these had multiple data collection time points [51,52,54,59].

### eHealth Programs and Patient Groups

Of the 12 included studies, 5 targeted the use of online clinical monitoring programs including patient-provider communication [48,52,53,55,59]. Three studies targeted use of ICBT [51,56,58]. Two used video consultations [49,54], one studied the implementation of both video consultation and ICBT [50], and one targeted online personal health records [57].

Four of the 12 studies included patients with somatic illnesses (chronic obstructive pulmonary disease, chronic heart failure, and chronic pain) [48,53,55,56], 3 studies included patients with mental health challenges (anxiety and depression) [50,51,58], and 5 studies included patients with long-term illnesses in general [49,52,54,57,59].

### Implementation Frameworks and Models

Of the total 12 studies, 8 used implementation frameworks or models to guide the analysis of implementation strategies and/or implementation outcomes. Two studies used the reach effectiveness, adoption, implementation, maintenance framework [50,54], and 2 studies used the normalization process theory [52,58]. Other frameworks/models were used by one study each: consolidated framework for implementation research [51], structurationism [49], promoting action on research implementation in health services [54] and the plan do study act cycle [55]. Finally, one study used the theoretical domains framework in combination with the technology acceptance model [56]. See Table 4 for details.

**Table 4:** Overview of included studies.

First author	Patient groups	eHealth	Setting	Implementation project	Implementation framework	Implementation stage	Study design	Data collection
Bailey [48]	COPD^a^	Clinical monitoring	Sheltered housing	4 tenants used telehealth for 16 weeks	N/A^b^	Middle^c^	Case study (QUAL^d^)	Self-report assessment, observation, focus groups, interviews, workshops
Boonstra [49]	Long-term illnesses	Video consultation	Homecare	From a database of 11,000 regular customers in 2006, 36 used the system	Structurationism	Middle	Single case study (MIXED)	Interviews, workshops, written reports, policy plans, meeting minutes, observations, quantitative data on system use
Fortney [50]	Depression	ICBT^e^, *Beating the Blues*, video consultation	Primary care safety net clinics	Implement EBP^f^ in 6 federally qualiﬁed health centers	RE-AIM^g^	Early^h^	Quality improvement methods (QUAL)	Qualitative needs assessments
Hadjistavropoulos [51]	Anxiety, depression	ICBT, *Wellbeing Course*	Community mental health clinics	ICBT implementation in 7 community mental health clinics	CFIR^i^	Late^j^	Process evaluation (QUANT^k^)	Online survey
Hendy [52]	Long-term illnesses	Clinical monitoring, *WSD*^l^	Primary care trusts	Case studies of 3 sites forming the WSD program	NPT^m^	Late	Comparative, longitudinal, qualitative, ethnographic case study (QUAL)	Interviews, meeting observations, document review
Hendy [59]	Long-term illnesses	Clinical monitoring, *WSD*	Health and social care organizations	Case studies representing 5 large public sector health organizations	N/A	Late	Longitudinal, ethnographic case studies (QUAL)	Observations, document review, informal discussions, interviews
Horton [53]	COPD	Clinical monitoring	Homecare	During the 6-month implementation period, only 10 users had been recruited to the scheme	N/A	Middle	Case study (QUAL)	Focus groups, field notes, meeting minutes
Lindsay [54]	PTSD^n^, anxiety, depression, insomnia, chronic pain, SUD^o^	Video consultation, *Video to Home*	VA^p^ Medical Center	This 2-year project included 93 patients	PARIHS^q^, RE-AIM	Late	Mixed-method program evaluation (MIXED)	Interviews, quantitative data on system use
Taylor [55]	COPD, chronic HF^r^	Clinical monitoring	Community health care	4 community nursing settings involved in 7-month program of action research	PDSA^s^	Middle	Case studies and action research methodologies (QUAL)	Workshop observations, focus groups, document review, field notes
Terpstra [56]	Chronic pain	ICBT, *Master Your Pain*	Mental health care institutions	13 mental health care institutions	TDF^t^, TAM^u^	Early	Descriptive design (QUANT)	Evaluation questionnaire
Wells [57]	Chronic illness	Online PHR^v^	Health delivery organizations	Health care organizations that had had a PHR in place for at least 12 months	N/A	Late	Grounded theory inductive approach (MIXED)	Interviews, Web-based survey
Wilhelmsen [58]	Depression	ICBT, *Mood-GYM*	General practice	3-day training package for GPs^w^ on ICBT	NPT	Late	Qualitative study (QUAL)	Telephone interviews

^a^COPD: chronic obstructive pulmonary disease.

^b^N/A: not applicable.

^c^Middle: 4-12 months postimplementation startup.

^d^QUAL: qualitative.

^e^ICBT: internet-delivered cognitive behavioral therapy.

^f^EBP: evidence-based practice.

^g^RE-AIM: reach, effectiveness, adoption, implementation, maintenance framework.

^h^Early: 0-3 months postimplementation startup.

^i^CFIR: consolidated framework for implementation research.

^j^Late: >12 months postimplementation startup.

^k^QUANT: quantitative.

^l^WSD: Whole Systems Demonstrator.

^m^NPT: normalization process theory.

^n^PTSD: posttraumatic stress disorder.

^o^SUD: substance use disorder.

^p^VA: Veterans Affairs.

^q^PARIHS: promoting action on research implementation in health services.

^r^HF: heart failure.

^s^PDSA: plan, do, study, act.

^t^TDF: theoretical domains framework.

^u^TAM: technology acceptance model.

^v^PHR: patient health record.

^w^GP: general practitioner.

### Implementation Strategies Reported

#### Overview

Nine of the 12 included studies reported the use of an overarching implementation strategy such as training [48,56,58], external facilitation [50,51,54], managerial strategies [59], action research [55], or a mixture of several discrete strategies [57]. Three studies did not describe any overarching implementation strategy, only describing the discrete strategies used [49,52,53].

When sorted according to the ERIC categories [22], 5 of the 12 studies reported implementation strategies within 7 or 8 categories [49-51,55,57], 2 reported implementation strategies within 5 or 6 categories [52,54], 2 reported implementation strategies within 3 or 4 categories [48,59], and 3 reported implementation strategies within 1 or 2 categories [53,56,58].

The category of implementation strategies most frequently reported was train and educate stakeholders (n=10), followed by change infrastructure (n=8), develop stakeholder interrelationships (n=8), use evaluative and iterative strategies (n=7), engage consumers (n=6), adapt and tailor to the context (n=5), use financial strategies (n=5), support clinicians (n=5), and finally provide interactive assistance (n=4). See Table 5 for details.

**Table 5:** Categories of implementation strategies [22] used in the included studies.

Studies	Engage consumers	Use evaluative and iterative strategies	Change infrastructure	Adapt and tailor to the context	Develop stakeholder interrelationships	Use financial strategies	Support clinicians	Provide interactive assistance	Train and educate stakeholders	Total categories reported	Overarching implementation strategy (study authors’ description)
Bailey [48]	x		x						x	3	Training
Boonstra [49]	x	x	x	x	x	x	x			7	Not reported
Fortney [50]	x	x	x	x	x			x	x	7	External facilitation/mixed
Hadjistavropoulos [51]	x	x			x	x	x	x	x	7	External facilitation
Hendy [52]		x	x		x		x		x	5	Not reported
Hendy [59]			x		x	x		x		4	Managerial strategies
Horton [53]			x						x	2	Not reported
Lindsay [54]		x	x	x	x			x	x	6	External facilitation
Taylor [55]	x	x	x	x	x	x	x		x	8	Action research
Terpstra [56]									x	1	Training
Wells [57]	x	x		x	x	x	x		x	7	Mixed
Wilhelmsen [58]									x	1	Training
Total	6	7	8	5	8	5	5	4	10		

#### Engage Consumers

Six of the 12 studies reported trying to reach and engage patients as one of their implementation strategies. This included advertising about the eHealth program to patients within their own institutions and/or to the wider community by means of newsletters, webpages, television, radio, newspapers, and direct contact with patients [49-51,57]. Other implementation strategies reported used to engage patients were inclusion of patients in research activities [55] and technical setup and support for patients in their homes [48,49].

#### Use Evaluative and Iterative Strategies

Seven of the 12 studies reported use of different evaluative and iterative strategies, either as stand-alone strategies or in combination with other strategies. Three of the 12 studies had made an implementation plan [50,55,57], 1 study had a business plan [49], and 1 study had included the eHealth implementation in the organizational vision statement [57]. Three studies focused on readiness, barriers, and facilitators [50,51,54]. Five studies reported that they made use of multiple stakeholder teams [50-52,55,57], and 1 study reported support from local clinical champions [50]. Five of the 12 studies reviewed the implementation progress [51,52,54,55,57], and 4 of them provided audit and feedback by feeding the information about the implementation progress back to the clinicians [51,54,55,57].

#### Change Infrastructure

Eight of the 12 studies reported purchase or acquisition of new electronic equipment as an implementation strategy [48-50,52-55,59].

#### Adapt and Tailor to the Context

Four of the 12 studies reported that they had cooperated with clinical staff to ensure tailoring of the eHealth program to meet local needs and organizational capabilities [50,54,55,57]. One study had cooperated with involved stakeholders to obtain a consistent implementation plan [49].

#### Develop Stakeholder Interrelationships

Four of the 12 studies reported involving multiple stakeholder teams at the overall management level, including representatives of the participating organizations such as care delivery organizations, telecom ﬁrms, insurance firms, commissioners, and industry [49,51,55,57]. Onsite project teams were established in 4 of the 12 studies [49,52,55,57]. Onsite clinical champions supported and promoted adoption of the eHealth program in 5 studies [50,52,54,55,57]. Management support and endorsement were reported in 3 studies [52,57,59]. One study also had visited other clinics to discuss concerns and impart their knowledge and experience [57].

#### Use Financial Strategies

Five of the 12 studies reported that they had used financial strategies related to the funding of the implementation projects [49,51,52], future cost-effectiveness aspects [49], and future financial investment aspects [55]. Incentives directed toward physicians’ performance indicators and monetary incentives and the use of gift card bonuses for clinicians were reported [57].

#### Support Clinicians

Four of the 12 studies had supported clinicians by recruiting new staff, establishing new roles, and supporting work process redesign [49,52,55,57]. Reminders to clinicians to prompt them to use the new eHealth programs were also reported [51].

#### Provide Interactive Assistance

Four of the 12 studies reported that they had used external researchers, consultants, or practitioners to provide external facilitation in terms of problem solving and support [50-52,54]. Training for local superusers was also reported conducted [54].

#### Train and Educate Stakeholders

Ten of the 12 studies reported that they had conducted training and teaching for clinicians about the eHealth programs being implemented. The education was reported as containing aspects related to the delivery of the clinical programs via eHealth [48,50-58], as well as technical aspects related to the eHealth software [48,51,57]. Six studies reported on the length of training and described a wide variety of time span, ranging from 2 to 3 hours [48,50,53] to 1 to 3 days [51,56,58].

### Implementation Outcomes Reported

#### Overview

All the 12 included studies reported implementation outcomes, ranging from 1 to 6 in each study. The 3 most frequently reported were acceptability, penetration, and adoption. See Table 6 for details on implementation strategies used and implementation outcomes reported.

**Table 6:** Implementation strategies used and implementation outcomes reported in the included studies.

First author	Implementation strategies		Implementation outcomes									Implementation success
	Categories of implementation strategies used	n	Acceptability	Adoption	Appropriateness	Cost	Feasibility	Fidelity	Penetration	Sustainability	n	Study authors’ evaluation of implementation success in relation to implementation strategies used
Bailey [48]	Engage consumers, change infrastructure, train and educate stakeholders	3	**+/–** ^a^	**+/–**	N/A^b^	N/A	N/A	N/A	N/A	N/A	2	Successful due to training and follow-up support
Boonstra [49]	Engage consumers, use evaluative and iterative strategies, change infrastructure, adapt and tailor to the context, use financial strategies, support clinicians, train and educate stakeholders	7	**+/–**	–^c^	–	N/A	–	N/A	–	N/A	5	Unsuccessful due to limited managerial agency and inconsistencies in some of the choices made during implementation phase
Fortney [50]	Engage consumers, use evaluative and iterative strategies, change infrastructure, adapt and tailor to the context, provide interactive assistance, train and educate stakeholders	7	N/A	N/A	N/A	N/A	N/A	N/A	**+/–**	**+** ^d^	2	Variable success across sites
Hadjistav-ropoulos [51]	Engage consumers, use evaluative and iterative strategies, develop stakeholder interrelationship, use financial strategies, support clinicians, provide interactive assistance, train and educate stakeholders	7	**+**	–	N/A	**+/–**	–	N/A	–	N/A	5	Successful due to ICBT^e^ program, implementation processes, and external facilitation. Could have been even better if planned in advance, all staff in the health region were informed about ICBT, and more resources were available
Hendy [52]	Use evaluative and iterative strategies, change infrastructure, develop stakeholder interrelationship, support clinicians, train and educate stakeholders	5	N/A	–	N/A	N/A	N/A	N/A	**+/–**	–	3	Unsuccessful despite resources deployed
Hendy [59]	Change infrastructure, develop stakeholder interrelationship, use financial strategies, provide interactive assistance	4	N/A	N/A	N/A	N/A	–	N/A	N/A	N/A	1	Unsuccessful due to lack of trust in individual managers
Horton [53]	Change infrastructure, train and educate stakeholders	2	–	N/A	N/A	N/A	N/A	N/A	N/A	N/A	1	Unsuccessful despite training and follow-up support
Lindsay [54]	Use evaluative and iterative strategies, change infrastructure, adapt and tailor to the context, develop stakeholder interrelationship, provide interactive assistance, train and educate stakeholders	6	**+**	N/A	N/A	**+**	–	–	**+**	**+**	6	Successful due to implementation facilitation strategy involving external and internal facilitators, especially clinical champions and training
Taylor [55]	Engage consumers, use evaluative and iterative strategies, change infrastructure, adapt and tailor to the context, develop stakeholder inter-relationship, use financial strategies, support clinicians, train and educate stakeholders	8	N/A	N/A	N/A	**+/–**	N/A	N/A	N/A	N/A	1	Mixed: 2 sites discontinued after first cycle because of competing priorities; positive experience of external facilitation by researchers and telehealth champions
Terpstra [56]	Train and educate stakeholders	1	**+**	N/A	N/A	N/A	N/A	N/A	N/A	N/A	1	N/A
Wells [57]	Engage consumers, use evaluative and iterative strategies, adapt and tailor to the context, develop stakeholder interrelationship, use financial strategies, support clinicians, train and educate stakeholders	7	**+**	N/A	N/A	N/A	N/A	N/A	**+/–**	N/A	2	Successful organizations actively communicated their vision; engaged leaders at all levels; had clear governance, planning, and protocols; set targets; and celebrated achievement. The most effective strategy for patient uptake was through health professional encouragement
Wilhelmsen [58]	Train and educate stakeholders	1	**+**	N/A	N/A	N/A	N/A	N/A	N/A	N/A	1	Not successful due to lack of practical training of module follow-ups in the course
Total			8	4	1	3	4	1	6	3		

^a^Mixed/neutral outcomes.

^b^Not applicable.

^c^Negative outcomes.

^d^Positive outcomes.

^e^ICBT: internet-delivered cognitive behavioral therapy.

#### Acceptability

Four of the 12 studies reported that health care providers had shown positive attitudes toward the eHealth program implemented [54,56-58]. One study reported low acceptability of their intervention [53]. Three studies reported mixed attitudes in that some were positive and some experienced the new eHealth program as a threat to or disturbance of their work [48,49,51].

#### Adoption

Four of the 12 studies reported challenges regarding the adoption of the eHealth programs into their clinical practice, describing difficulties motivating the clinicians to approach their clients with the new eHealth program [49,51-53]. Time available and time frame given were also reported to pose organizational challenges [48,52]. None of the included studies presented solely positive descriptions of the adoption of the eHealth programs.

#### Appropriateness

Only 1/12 included studies reported on appropriateness, stating that the technology might not always be appropriate, for example, if advanced age, poverty, or serious illnesses might amplify the clients’ vulnerability [49].

#### Cost

Three studies mentioned costs. One study reported no additional costs related to the eHealth implementation [51], 1 study reported travel expenditures saved [54], and 1 study described being unable to calculate costs due to lack of robust data [55].

#### Feasibility

Four of the 12 studies reported low feasibility for their eHealth programs [49,51,52,54], describing the innovations as an interruption to the real work and as difficult to integrate with existing patient workloads.

#### Fidelity

Fidelity was reported in only 1 of the 12 studies, stating that high fidelity was difficult to achieve due to providers’ need to remain flexible and the program needed to be adapted to the technology platform already present in the clinical setting [54].

#### Penetration

Four studies presented how many patients received an eHealth program [50,52,54,57], only one of which reported satisfaction with how many patients received the program [54]. Two studies indicated limited numbers of patients who received the eHealth program being studied, but did not provide exact figures [49,51].

#### Sustainability

Three of the 12 included studies reported sustainability. In two instances, the eHealth programs were sustained after the implementation efforts [50,54], while the third did not achieve sustainability [52].

### Implementation Success Reported

All studies except one [56] reported on implementation success. The majority provided a direct [48,50,52,57-59] or indirect [49,53,54] description of how they defined implementation success. This spanned from concrete definitions such as “the number of people in each site using the new service” [59] to more vague descriptions such as “change in terms of telecare appropriation was realized” [49]. As the assessment of implementation success was used as a means to evaluate the reported implementation outcomes in this review, and implementation success is often derived directly from the implementation outcomes, the two aspects (ie, success and outcome) were not necessarily mutually exclusive. Four studies reported that the implementation had been successful [48,51,54,57], while 5 studies reported unsuccessful implementation [49,52,53,58,59]. Two studies reported mixed results, with implementation being successful at some of the sites and unsuccessful at the others [50,55].

### Relationship Between Implementation Strategies, Implementation Outcomes, and Implementation Success

In the 12 included studies, no relationship was detected between implementation strategies [22] and implementation outcomes [37].

Regarding implementation success, the implementation strategies management support and engagement, internal and external facilitation, training, and audit and feedback were directly related to implementation success in several studies. For example, management support and engagement were highlighted as important for implementation success in 1 study [57], and lack of trust or limited managerial agency was described as a contributing factor to implementation failure in 2 other studies [49,59]. Furthermore, external facilitation was reported to be important for implementation success in 4 studies [50,51,54,55]. Internal facilitation, especially the support and engagement of clinical or implementation champions, was highlighted as important for the implementation success in 2 studies [54,55]. In addition, training and education of stakeholders were used as implementation strategies in studies reporting successful [48,54,56] as well as unsuccessful implementation [53,58].

No clear relationship was found between the number of implementation strategies used and implementation success. For example, of 3 studies using a range of implementation strategies, 1 reported implementation success [51], 1 reported implementation failure [49], and 1 reported mixed results [55]. Furthermore, of 2 studies using training and education of stakeholders as the only implementation strategy, 1 reported implementation success [56] and 1 reported implementation failure [58]. There was no relationship between reported implementation success and use of implementation frameworks.

## Discussion

### Summary of Evidence and Comparison With Prior Work

This systematic realist review used the categorization of implementation strategies by the ERIC taxonomy [21,22] and the implementation outcome framework by Proctor and colleagues [37] as data extraction templates. As no specific framework exists for implementation success, this was qualitatively summarized based on the study authors’ own definition. The review identified and synthesized 12 studies examining implementation strategies, implementation outcomes, and implementation success related to the implementation of eHealth programs for patients with chronic illnesses. Findings show that there has so far been little focus on reporting implementation strategies for eHealth implementation where the patient is the main user of the program. Also, there appears to be great variety in implementation terms used and considerable vagueness in the description of implementation aspects, which led the authors to have to screen a number of irrelevant full-text studies. There were also challenges in the data extraction process due to inconsistence in terminology used in the studies. Other researchers have also pointed to inconsistencies in use of terminology and definitions related to implementation [20,60,61]. Due to great heterogeneity in the included studies with regard to types of patient conditions, eHealth interventions, and phases of implementation, it was not possible to detect any relationship between these factors related to implementation strategies, implementation outcomes, and implementation success.

A wide range of implementation strategies were used in the studies included in the review. The most frequently used categories of implementation strategies were train and educate stakeholders, change infrastructure, and develop stakeholder interrelationships. Included in the latter category is involvement of champions, which has also been identified as central to implementation success by other reviews [62,63]. Several of the included studies reported training of health care personnel as a preferred implementation strategy, and this strategy was also found to be widely used by others, even though effects appear inconsistent [62,64]. Despite recent evidence pointing to tailored implementations as effective [62,65], only 4 studies in the review reported that they had tailored the eHealth intervention to meet the context where the implementation took place. Also, several frameworks for technology implementation have pointed to the importance of contextual factors as key elements to address in order to succeed, including the CeHRes (Center for eHealth Research and Disease Management) roadmap [66] and the NASSS framework [28]. The limited use of tailoring so far in the implementation context could potentially be one explanation for the limited implementation success to date.

Implementation outcomes were reported in all 12 studies included in this review, with each individual study reporting between 1 and 6 implementation outcomes. The implementation outcomes most frequently reported were acceptability and penetration. As the included studies had not aimed to report on implementation outcomes, only a few of the terms in the implementation outcome framework [37] were covered. It is thus reasonable to assume that implementation outcomes were underreported in many of the included studies. Based on this, it was not possible to detect any clear relationship between implementation strategies and implementation outcomes in the review. However, it might not be a coincidence that these 12 studies that reported implementation strategies also reported implementation outcomes. Because when people really start to think about and report implementation strategies, they will also think about reporting at least some implementation outcomes. In order to still allow for an evaluation of how successful the implementation had been when the implementation outcome framework was not suitable enough for a mechanism evaluation, implementation success was also included in this equation.

Regarding implementation success, 4 of the included 12 studies reported success, 5 reported lack of success, and 2 reported mixed results. Training and education of stakeholders showed mixed relations to implementation success, indicating that the content, duration, and facilitation of the training are important for training effectiveness. The studies offering the most training are not necessarily the most successful, indicating that other factors (eg, clinician motivation and intention to use the new eHealth program) also play an important role [4]. This review suggests that a combination of software training and training in how to use the technology in daily work may be necessary. These findings are in line with other reviews that have also highlighted training, support, and supervision as key factors in order for clinicians to start using new eHealth programs [30,35]. Due to the limited coverage provided by the implementation outcome framework, as described above, no clear relationship between implementation outcomes and implementation success could be detected in the review. For example, one of the studies showed that the implementation can be successful or experienced as successful even with negative scores on some of the implementation outcomes concepts [54]. However, in more than half of the studies in the review, there was coherence between the ratings on implementation outcomes and implementation success [49,50,52,53,55,57,59]. Due to the limited number of implementation outcome concepts covered, however, this finding must be interpreted with caution. Given a more comprehensive reporting on implementation outcomes, the coherence could have been different. The relationship between implementation outcomes and implementation success still appears a conundrum. This has also been pointed out by others [37] and should be further investigated in future studies. Although not the topic of this review, it is also worth mentioning that if the patient outcomes (eg, effect of the intervention) do not occur, positive implementation outcomes and implementation success does not have much impact.

Another important finding from the review is that several studies showed the implementation strategies related to management engagement to be directly related to implementation success. Other researchers have found leadership to be crucial in order to succeed with implementation of evidence-based practice and have also pointed to the setting in which the leader operates as being of importance [67].

The successful implementation efforts identified in this review, reaching sustainability for more than 1 year after start-up [51,54,57], were all related to use of a mixture of several implementation strategies and were also supported by internal and external facilitation. All of these studies also provided audit and feedback, one of the implementation strategies with evidence for effectiveness [62,68].

No clear relationship was found in the review between the number of implementation strategies used and implementation success. The successful implementation projects described used multifaceted strategies. However, one study used a single strategy and was still successful [56]. This shows that the quality of an implementation strategy might be more important than the quantity, which is in line with a former review concluding that multifaceted strategies are not necessarily more effective than single strategies [64].

Despite the importance of describing and sharing information about unsuccessful implementations, the continued degree of unsuccessful implementation efforts is disturbing and gives cause for concern. It is, however, possible that the lack of a systematic implementation approach and the lack of employing proposed successful implementation strategies can provide explanation for this challenge.

Finally, the results from this review also indicate that reaching sustainability is and remains a challenge despite use and focus on implementation strategies.

### Implications for Research and Practice

This systematic realist review clearly demonstrates a need for more studies that report on implementation strategies, implementation outcomes, implementation success, and the relationship among these in eHealth implementation. The research on implementation strategies is still in its infancy, and more work is needed to better understand how implementation strategies can contribute to improved implementation effectiveness [23].

This review also demonstrates the need for implementation planning at a very early stage—that is, already in the design and development phase of eHealth support and intervention programs. Low feasibility of many of the eHealth programs included in this review clearly shows an urgent need to include all stakeholders in the early phases of program development. Also, implementation planning must be included from the very beginning in order to adapt interventions to context and enable implementation. As such, using frameworks for eHealth development and implementation, such as the CehRes roadmap [66] that combines aspects from human-centered design, persuasive technology, and business modeling, can help address implementation aspects already in the phase of idea generation and problem identification.

When planning and conducting eHealth implementation in clinical practice, evidence is still lacking about proposing clear advice on how implementation strategies can be used effectively when implementing eHealth programs to support patients in their own homes. This review concludes, in support of existing research, that the question of which implementation strategies are the most effective under which circumstances still remains unclear [64]. Nonetheless, this review indicates that internal and external facilitation, audit and feedback, management support, and training of clinicians are essential. Lacking more robust evidence on specific implementation strategies for eHealth implementation, general evidence on implementation strategies must be considered.

### Limitations

This systematic realist review has limitations that need to be considered when interpreting the results. First, in order to get a manageable number of hits from the literature search, some limitations to the search strategy were necessary. Therefore, the search was performed on published studies only since 2006. Prior to 2006, the eHealth and implementation research fields were both in their infancy and few publications were assumed to exist. This review process showed the earliest publication included to be from 2008, supporting this assumption. Therefore, no publications were included from the period 2006 to 2008. Another restriction intended to keep the hits to a manageable number was to limit the chronic illnesses included.

Use of predefined categories for data abstraction and analysis has strengths as well as limitations. In the review, the ERIC project [22] and the implementation outcome framework [37] were used to guide the review process. There is a potential risk that aspects not covered in the two categorizations could be overlooked in the review, as different frameworks provide different lenses through which research problems can be analyzed [69]. The ERIC categories are comprehensive and posed some challenges regarding overlap between categories. Furthermore, as not all included studies had implementation aspects as their only focus, the data extraction process could have introduced potential risks of overlooking or omitting aspects of implementation strategies, implementation outcomes, and implementation success. Inconsistent use of language and terminology in the 12 included studies also made it challenging to sort and label implementation strategies and outcomes. The validation process conducted by two authors nevertheless showed no discrepancy in data extraction.

### Conclusions

This is the first review examining implementation strategies, implementation outcomes, and implementation success of studies reporting on the implementation of eHealth programs for patients with chronic illnesses. Findings suggest that internal and external facilitation, management support, and training of clinicians are important factors for the success of eHealth implementation. The results also highlight the lack of eHealth studies reporting implementation strategies in a comprehensive way, pointing to the need for designing robust studies on implementation strategies in the future.

## Appendix

Multimedia Appendix 1 Search strategy.

## Abbreviations

eHealth: electronic health
CeHRes: Center for eHealth Research and Disease Management
ERIC: Expert Recommendations for Implementing Change
ICBT: internet-delivered cognitive behavioral therapy
mHealth: mobile health
NASSS: framework for nonadoption, abandonment, scale-up, spread, and sustainability
PROSPERO: Prospective Register of Systematic Reviews

## References

[1] Eysenbach G (2001). What is eHealth?. J Med Internet Res.

[2] Morton K, Dennison L, May C, Murray E, Little P, McManus RJ, Yardley L (2017). Using digital interventions for self-management of chronic physical health conditions: a meta-ethnography review of published studies. Patient Educ Couns.

[3] Mehta S, Peynenburg VA, Hadjistavropoulos HD (2018). Internet-delivered cognitive behaviour therapy for chronic health conditions: a systematic review and meta-analysis. J Behav Med.

[4] Varsi C, Ekstedt M, Gammon D, Ruland CM (2015). Using the consolidated framework for implementation research to identify barriers and facilitators for the implementation of an internet-based patient-provider communication service in five settings: a qualitative study. J Med Internet Res.

[5] Borosund E, Varsi C, Clark M, Ehlers S, Andrykowski M, Sleveland H (2019). Pilot testing an app-based stress management intervention for cancer survivors [in press]. Translat Behav Med.

[6] Harerimana B, Forchuk C, O'Regan T (2019). The use of technology for mental healthcare delivery among older adults with depressive symptoms: a systematic literature review. Int J Ment Health Nurs.

[7] Kruse CS, Beane A (2018). Health information technology continues to show positive effect on medical outcomes: systematic review. J Med Internet Res.

[8] Mold F, de Lusignin S, Sheikh A, Majeed A, Wyatt JC, Quinn T, Cavill M, Franco C, Chauhan U, Blakey H, Kataria N, Arvanitis TN, Ellis B (2015). Patients' online access to their electronic health records and linked online services: a systematic review in primary care. Br J Gen Pract.

[9] Whitehead L, Seaton P (2016). The effectiveness of self-management mobile phone and tablet apps in long-term condition management: a systematic review. J Med Internet Res.

[10] Hanlon P, Daines L, Campbell C, McKinstry B, Weller D, Pinnock H (2017). Telehealth interventions to support self-management of long-term conditions: a systematic metareview of diabetes, heart failure, asthma, chronic obstructive pulmonary disease, and cancer. J Med Internet Res.

[11] Brettle A, Brown T, Hardiker N, Radcliffe J, Smith C (2013). Telehealth: the effects on clinical outcomes, cost effectiveness and the patient experience: a systematic overview of the literature.

[12] Deady M, Choi I, Calvo RA, Glozier N, Christensen H, Harvey SB (2017). eHealth interventions for the prevention of depression and anxiety in the general population: a systematic review and meta-analysis. BMC Psychiatry.

[13] Thurnheer SE, Gravestock I, Pichierri G, Steurer J, Burgstaller JM (2018). Benefits of mobile apps in pain management: systematic review. JMIR Mhealth Uhealth.

[14] Turgoose D, Ashwick R, Murphy D (2018). Systematic review of lessons learned from delivering tele-therapy to veterans with post-traumatic stress disorder. J Telemed Telecare.

[15] Elbert NJ, van Os-Medendorp H, van Renselaar W, Ekeland AG, Hakkaart-van Roijen L, Raat H, Nijsten TEC, Pasmans SGMA (2014). Effectiveness and cost-effectiveness of ehealth interventions in somatic diseases: a systematic review of systematic reviews and meta-analyses. J Med Internet Res.

[16] Buntin MB, Burke MF, Hoaglin MC, Blumenthal D (2011). The benefits of health information technology: a review of the recent literature shows predominantly positive results. Health Aff (Millwood).

[17] Greenhalgh T, Shaw S, Wherton J, Vijayaraghavan S, Morris J, Bhattacharya S, Hanson P, Campbell-Richards D, Ramoutar S, Collard A, Hodkinson I (2018). Real-world implementation of video outpatient consultations at macro, meso, and micro levels: mixed-method study. J Med Internet Res.

[18] Ross J, Stevenson F, Lau R, Murray E (2016). Factors that influence the implementation of e-health: a systematic review of systematic reviews (an update). Implement Sci.

[19] Powell BJ, McMillen JC, Proctor EK, Carpenter CR, Griffey RT, Bunger AC, Glass JE, York JL (2012). A compilation of strategies for implementing clinical innovations in health and mental health. Med Care Res Rev.

[20] Proctor EK, Powell BJ, McMillen JC (2013). Implementation strategies: recommendations for specifying and reporting. Implement Sci.

[21] Powell BJ, Waltz TJ, Chinman MJ, Damschroder LJ, Smith JL, Matthieu MM, Proctor EK, Kirchner JE (2015). A refined compilation of implementation strategies: results from the Expert Recommendations for Implementing Change (ERIC) project. Implement Sci.

[22] Waltz TJ, Powell BJ, Matthieu MM, Damschroder LJ, Chinman MJ, Smith JL, Proctor EK, Kirchner JE (2015). Use of concept mapping to characterize relationships among implementation strategies and assess their feasibility and importance: results from the Expert Recommendations for Implementing Change (ERIC) study. Implement Sci.

[23] Powell BJ, Fernandez ME, Williams NJ, Aarons GA, Beidas RS, Lewis CC, McHugh SM, Weiner BJ (2019). Enhancing the impact of implementation strategies in healthcare: a research agenda. Front Public Health.

[24] Wensing M, Grol R (2019). Knowledge translation in health: how implementation science could contribute more. BMC Med.

[25] Nilsen P (2015). Making sense of implementation theories, models and frameworks. Implement Sci.

[26] Ritchie M, Dollar K, Miller C, Oliver K, Smith J, Lindsay J (2017). Quality Enhancement Research Initiative (QUERI) for Team-Based Behavioral Health.

[27] Bucknall T, Rycroft-Malone J, Bucknall T, Rycroft-Malone J (2010). Evidence-based practice: doing the right thing for patients. Models and Frameworks for Implementing Evidence-Based Practice: Linking Evidence to Action.

[28] Greenhalgh T, Wherton J, Papoutsi C, Lynch J, Hughes G, A'Court C, Hinder S, Fahy N, Procter R, Shaw S (2017). Beyond adoption: a new framework for theorizing and evaluating nonadoption, abandonment, and challenges to the scale-up, spread, and sustainability of health and care technologies. J Med Internet Res.

[29] Greenhalgh T, Wherton J, Papoutsi C, Lynch J, Hughes G, A'Court C, Hinder S, Procter R, Shaw S (2018). Analysing the role of complexity in explaining the fortunes of technology programmes: empirical application of the NASSS framework. BMC Med.

[30] Drozd F, Vaskinn L, Bergsund HB, Haga SM, Slinning K, Bjørkli CA (2016). The implementation of internet interventions for depression: a scoping review. J Med Internet Res.

[31] Hage E, Roo JP, van Offenbeek MA, Boonstra A (2013). Implementation factors and their effect on e-Health service adoption in rural communities: a systematic literature review. BMC Health Serv Res.

[32] Scott KC, Karem P, Shifflett K, Vegi L, Ravi K, Brooks M (2018). Evaluating barriers to adopting telemedicine worldwide: a systematic review. J Telemed Telecare.

[33] Mair FS, May C, O'Donnell C, Finch T, Sullivan F, Murray E (2012). Factors that promote or inhibit the implementation of e-health systems: an explanatory systematic review. Bull World Health Organ.

[34] Saliba V, Legido-Quigley H, Hallik R, Aaviksoo A, Car J, McKee M (2012). Telemedicine across borders: a systematic review of factors that hinder or support implementation. Int J Med Inform.

[35] Brewster L, Mountain G, Wessels B, Kelly C, Hawley M (2014). Factors affecting front line staff acceptance of telehealth technologies: a mixed-method systematic review. J Adv Nurs.

[36] O'Connor S, Hanlon P, O'Donnell CA, Garcia S, Glanville J, Mair FS (2016). Understanding factors affecting patient and public engagement and recruitment to digital health interventions: a systematic review of qualitative studies. BMC Med Inform Decis Mak.

[37] Proctor E, Silmere H, Raghavan R, Hovmand P, Aarons G, Bunger A, Griffey R, Hensley M (2011). Outcomes for implementation research: conceptual distinctions, measurement challenges, and research agenda. Adm Policy Ment Health.

[38] Pawson R, Greenhalgh T, Harvey G, Walshe K (2005). Realist review: a new method of systematic review designed for complex policy interventions. J Health Serv Res Policy.

[39] Rycroft-Malone J, McCormack B, Hutchinson AM, DeCorby K, Bucknall TK, Kent B, Schultz A, Snelgrove-Clarke E, Stetler CB, Titler M, Wallin L, Wilson V (2012). Realist synthesis: illustrating the method for implementation research. Implement Sci.

[40] Gough D (2013). Meta-narrative and realist reviews: guidance, rules, publication standards and quality appraisal. BMC Med.

[41] World Health Organization (2011). Global Observatory for eHealth Series.

[42] (2019). World Health Organization.

[43] (2019). What is a patient portal?.

[44] Rabin BA, Brownson RC, Haire-Joshu D, Kreuter MW, Weaver NL (2008). A glossary for dissemination and implementation research in health. J Public Health Manag Pract.

[45] Huynh AK, Hamilton AB, Farmer MM, Bean-Mayberry B, Stirman SW, Moin T, Finley EP (2018). A pragmatic approach to guide implementation evaluation research: strategy mapping for complex interventions. Front Public Health.

[46] Nielsen B, Slinning K, Weie Oddli H, Drozd F (2018). Identification of implementation strategies used for the circle of security-Virginia family model intervention: concept mapping study. JMIR Res Protoc.

[47] Perry CK, Damschroder LJ, Hemler JR, Woodson TT, Ono SS, Cohen DJ (2019). Specifying and comparing implementation strategies across seven large implementation interventions: a practical application of theory. Implement Sci.

[48] Bailey C, Cook G, Herman L, McMillan C, Rose J, Marston R, Binks E, Barron E (2015). Deploying telehealth with sheltered housing tenants living with COPD: a qualitative case study. Housing, Care and Support.

[49] Boonstra A, Van Offenbeek M (2010). Towards consistent modes of e-health implementation: structurational analysis of a telecare programme's limited success. Info Systems J.

[50] Fortney JC, Pyne JM, Ward-Jones S, Bennett IM, Diehl J, Farris K, Cerimele JM, Curran GM (2018). Implementation of evidence-based practices for complex mood disorders in primary care safety net clinics. Fam Syst Health.

[51] Hadjistavropoulos HD, Nugent MM, Dirkse D, Pugh N (2017). Implementation of internet-delivered cognitive behavior therapy within community mental health clinics: a process evaluation using the consolidated framework for implementation research. BMC Psychiatry.

[52] Hendy J, Chrysanthaki T, Barlow J, Knapp M, Rogers A, Sanders C, Bower P, Bowen R, Fitzpatrick R, Bardsley M, Newman S (2012). An organisational analysis of the implementation of telecare and telehealth: the whole systems demonstrator. BMC Health Serv Res.

[53] Horton K (2008). The use of telecare for people with chronic obstructive pulmonary disease: implications for management. J Nurs Manag.

[54] Lindsay JA, Hudson S, Martin L, Hogan JB, Nessim M, Graves L, Gabriele J, White D (2017). Implementing video to home to increase access to evidence-based psychotherapy for rural veterans. J Technol Behav Sci.

[55] Taylor J, Coates E, Wessels B, Mountain G, Hawley MS (2015). Implementing solutions to improve and expand telehealth adoption: participatory action research in four community healthcare settings. BMC Health Serv Res.

[56] Terpstra JA, van der Vaart R, Spillekom-van Koulil S, van Dam A, Rosmalen JGM, Knoop H, van Middendorp H, Evers AWM (2018). Becoming an eCoach: training therapists in online cognitive-behavioral therapy for chronic pain. Patient Educ Couns.

[57] Wells S, Rozenblum R, Park A, Dunn M, Bates DW (2015). Organizational strategies for promoting patient and provider uptake of personal health records. J Am Med Inform Assoc.

[58] Wilhelmsen M, Høifødt RS, Kolstrup N, Waterloo K, Eisemann M, Chenhall R, Risør MB (2014). Norwegian general practitioners' perspectives on implementation of a guided web-based cognitive behavioral therapy for depression: a qualitative study. J Med Internet Res.

[59] Hendy J, Chrysanthaki T, Barlow J (2014). Managers’ identification with and adoption of telehealthcare. Societies.

[60] Eldh AC, Almost J, DeCorby-Watson K, Gifford W, Harvey G, Hasson H, Kenny D, Moodie S, Wallin L, Yost J (2017). Clinical interventions, implementation interventions, and the potential greyness in between: a discussion paper. BMC Health Serv Res.

[61] Leeman J, Birken SA, Powell BJ, Rohweder C, Shea CM (2017). Beyond “implementation strategies”: classifying the full range of strategies used in implementation science and practice. Implement Sci.

[62] Pantoja T, Opiyo N, Lewin S, Paulsen E, Ciapponi A, Wiysonge CS, Herrera CA, Rada G, Peñaloza B, Dudley L, Gagnon M, Garcia Marti S, Oxman AD (2017). Implementation strategies for health systems in low-income countries: an overview of systematic reviews. Cochrane Database Syst Rev.

[63] Shea CM, Belden CM (2016). What is the extent of research on the characteristics, behaviors, and impacts of health information technology champions? A scoping review. BMC Med Inform Decis Mak.

[64] Lau R, Stevenson F, Ong BN, Dziedzic K, Treweek S, Eldridge S, Everitt H, Kennedy A, Qureshi N, Rogers A, Peacock R, Murray E (2015). Achieving change in primary care—effectiveness of strategies for improving implementation of complex interventions: systematic review of reviews. BMJ Open.

[65] Baker R, Camosso-Stefinovic J, Gillies C, Shaw EJ, Cheater F, Flottorp S, Robertson N, Wensing M, Fiander M, Eccles MP, Godycki-Cwirko M, Jäger C (2015). Tailored interventions to address determinants of practice. Cochrane Database Syst Rev.

[66] van Gemert-Pijnen JE, Nijland N, van Limburg M, Ossebaard HC, Kelders SM, Eysenbach G, Seydel ER (2011). A holistic framework to improve the uptake and impact of eHealth technologies. J Med Internet Res.

[67] Sandström B, Borglin G, Nilsson R, Willman A (2011). Promoting the implementation of evidence-based practice: a literature review focusing on the role of nursing leadership. Worldviews Evid Based Nurs.

[68] Ivers NM, Grimshaw JM, Jamtvedt G, Flottorp S, O'Brien MA, French SD, Young J, Odgaard-Jensen J (2014). Growing literature, stagnant science? Systematic review, meta-regression and cumulative analysis of audit and feedback interventions in health care. J Gen Intern Med.

[69] Reeves S, Albert M, Kuper A, Hodges BD (2008). Why use theories in qualitative research?. BMJ.

