# Supplementary material for: Implementation Strategies to Enhance the Implementation of eHealth Programs for Patients With Chronic Illnesses: Realist Systematic Review
Source: J Med Internet Res. 2019 Sep 27;21(9):e14255. doi: 10.2196/14255 (PMC6789428; doi:10.2196/14255)
Supplement: Multimedia Appendix 1 [file jmir_v21i9e14255_app1.pdf]

Multimedia Appendix 1. Search strategy.

Ovid MEDLINE(R) Epub Ahead of Print, In-Process & Other Non-Indexed Citations, Ovid MEDLINE(R) Daily and Ovid MEDLINE(R) <1946 to Present>

Search conducted 9 November 2017 and updated 4 October 2018 and modified for each database.

1	exp diffusion of innovation/
2	exp Technology Transfer/
3	exp Translational Medical Research/
4	exp Organizational Innovation/
5	exp Health Plan Implementation/
6	Information Dissemination/
7	implement*.tw,kf.
8	dissemin*.tw,kf.
9	(adoption or adopter or adopters or adaptation or adapt).tw,kf.
10	(organi?ational innovation* or organi?ational change*).tw,kf.
11	(diffuse or diffusion).tw,kf.
12	((system or systems or systematic or systematically) adj2 (change or changer or changed or changes)).tw,kf.
13	uptake*.tw,kf.
14	sustainab*.tw,kf.
15	institutionali*.tw,kf.
16	routin*.tw,kf.
17	maintenance.tw,kf.
18	incorporat*.tw,kf.
19	integrat*.tw,kf.
20	(Implement* adj3 fidelity).tw,kf.
21	quality improvement*.tw,kf.
22	Facilitat*.tw,kf.
23	Barrier*.tw,kf.

24	((knowledge or technolog* or research) adj3 (transform* or translat* or exchange or transfer or integration or utilization)).tw,kf.
25	or/1-24
26	Therapy, Computer-Assisted/ or Drug Therapy, Computer-Assisted/
27	Telemedicine/
28	Telerehabilitation/
29	exp Internet/ and (health or counsel?ing or coach* or rehabilitat* or communicat* or support* or follow up or therap* or intervention? or treatment* or interaction?).tw,kf.
30	Mobile Applications/ and (health or counsel?ing or coach* or rehabilitat* or communicat* or support* or follow up or therap* or intervention? or treatment* or interaction?).tw,kf.
31	Telecommunications/ and (health or counsel?ing or coach* or rehabilitat* or communicat* or support* or follow up or therap* or intervention? or treatment* or interaction?).tw,kf.
32	(Smartphone/ or Cell Phones/) and (health or counsel?ing or coach* or rehabilitat* or communicat* or support* or follow up or therap* or intervention? or treatment* or interaction?).tw,kf.
33	Computers, Handheld/ and (health or counsel?ing or coach* or rehabilitat* or communicat* or support* or follow up or therap* or intervention? or treatment* or interaction?).tw,kf.
34	Telenursing/
35	((Tele adj rehabilitation*) or Telerehabilitation*).tw,kf.
36	(Telehealth or (Tele adj health*)).tw,kf.
37	(eHealth or ehealth or e-therap* or "etherap*" or "e-mental" or emental or m-health or mhealth or (mobile adj health) or Telemedic* or telecommunicat* or teleconsult* or telecounsel?ing or teleconferenc* or telecoach* or (Tele adj home) or Telehome* or (Tele adj care) or telecare*).tw,kf.
38	((telephone* or phone* or cellphone* or smartphone* or ((cell or mobile or smart) adj2 phone*) or mobile or tablet or tablets or "internet delivered" or "internet based" or "internet mediated" or "internet supported" or online or web-based or webbased or "web page" or "web application" or website? or application? or app or apps or "Medical informatics" or "Information technology" or webcamera? or web-camera? or tele* or remote* or distan*) adj3 (counsel?ing or coach* or rehabilitat* or communicat* or support* or follow up or therap* or intervention? or treatment* or interaction?)).tw,kf.
39	or/26-38
40	exp Chronic Disease/ or ((chronic adj3 (illness or disease?)) or chronicity).tw,kf.

41	exp Arthritis, Rheumatoid/ or (Osteoarthritis or arthritis or spondyl*).tw,kf.
42	Chronic Pain/ or (chronic adj3 (pain or pains or painful)).tw,kf.
43	pulmonary disease, chronic obstructive/ or (chronic obstructive pulmonary disease or copd).tw,kf.
44	obesity/ or obesity hypoventilation syndrome/ or obesity, abdominal/ or obesity, metabolically benign/ or obesity, morbid/ or (obese or obesity or overweight).tw,kf.
45	diabetes mellitus/ or exp diabetes mellitus, type 1/ or exp diabetes mellitus, type 2/ or diabetic ketoacidosis/ or donohue syndrome/ or latent autoimmune diabetes in adults/ or prediabetic state/ or (diabetes or diabetic*).tw,kf.
46	Anxiety/ or exp Anxiety Disorders/ or Anxiety, Separation/ or Depression/ or Depressive Disorder/ or Depression, involutional/ or Dysthymic disorder/ or Seasonal affective disorder/ or Bereavement/
47	(nervousness or anxiety or anxiousness or angst or apprehension or fear).tw,kf.
48	(obsessive compulsive disorder$ or obsessive compulsive neuros?s or compulsive neuros?s or obsessive neuros?s).tw,kf.
49	(phobic disorder$ or phobic neuros?s or phobia$).tw,kf.
50	(stress disorder$ or posttraumatic neuros?s or post traumatic neuros?s).tw,kf.
51	(depression or depressive or depressed or melanchol* or dysphoria or despair or despondency).tw,kf.
52	(seasonal affective disorder$ or seasonal mood disorder$).tw,kf.
53	(dysthymic disorder$ or dysthymia).tw,kf.
54	(sadness or low mood).tw,kf.
55	panic.tw,kf.
56	Panic/
57	(Social Phobia? or Social Anxiety Disorder?).tw,kf.
58	or/40-57
59	25 and 39 and 58
60	limit 59 to (danish or english or norwegian or swedish or dutch)
61	limit 60 to yr="2006 -Current"
62	limit 61 to (addresses or autobiography or bibliography or biography or comment or congresses or consensus development conference or consensus development conference, nih or dataset or dictionary or directory or editorial or festschrift or historical article or interactive tutorial or interview or lectures or letter or news or newspaper article or personal narratives or portraits or technical report or twin study or validation studies or video-audio media)

63	61 not 62
